# *Radula rheophila*, a new species of *Radula* (Radulaceae) from Thailand and Brunei Darussalam

**DOI:** 10.3897/phytokeys.271.181229

**Published:** 2026-03-06

**Authors:** Chatchaba Promma, Rui-Liang Zhu, Sahut Chantanaorrapint

**Affiliations:** 1 PSU-Herbarium, Division of Biological Science, Faculty of Science, Prince of Songkla University, Hat Yai, Songkhla 90110, Thailand Department of Biology, School of Life Sciences, East China Normal University Shanghai China; 2 Bryology Laboratory, Department of Biology, School of Life Sciences, East China Normal University, 500 Dongchuan Road, Shanghai 200241, China Faculty of Science, Prince of Songkla University Songkhla Thailand

**Keywords:** Biodiversity hotspot, liverworts, molecular phylogeny, new taxon, *Radula* subg. *Odontoradula*, rheophyte

## Abstract

During a revision of Radulaceae in Southeast Asia, an undescribed species of the genus *Radula* was discovered from Thailand and Brunei Darussalam. Morphological characters were examined from fresh and herbarium specimens using stereo and compound microscopes. Phylogenetic analyses were conducted based on six chloroplast markers (*atp*B*–rbc*L, *psb*T*–psb*H, *psb*A*–trn*H, *rps*4, *trn*G, and *trn*L–F) using Maximum Likelihood and Bayesian Inference approaches. *Radula rheophila* is described and illustrated as a new species from peninsular Thailand and Brunei Darussalam. The new species is characterized by female bracts usually in 2 or 2.5 pairs unequal pairs; longitudinally rectangular or ligulate leaf lobules when large, broadly transversely rhombic or rectangular when small, 1/3–2/5 the length of the lobe; ovate to broadly ovate leaf lobes with broadly obtuse to rounded apices and entire margins; finely botryoidal oil bodies (2–3 per cell); and stems with thickened exterior cortical walls and thin-walled interior cortical and medullary cells thickened at the corners by concave trigones. Phylogenetic analyses strongly support its placement within *Radula* subg. *Odontoradula*. The discovery of *R. rheophila* enhances understanding of morphological variation and phylogenetic relationships within *Radula*, highlighting the significance of integrative taxonomy for uncovering tropical bryophyte diversity.

## Introduction

*Radula* Dumort. is the largest genus of the family Radulaceae, comprising approximately 242 currently accepted species (Promma et al. 2025). The genus exhibits a cosmopolitan distribution, with its greatest diversity occurring in wet tropical and subtropical regions. Members of *Radula* are easily recognized by several distinctive features, including rhizoids arising from the lobules rather than the stem, the absence of underleaves on the ventral merophyte, *Radula*-type branching, the presence of 1(–6) large oil bodies per leaf cell, and dorso-ventrally compressed perianths.

Based on previous molecular phylogenetic studies, the infrageneric classification of *Radula* was originally divided into seven subgenera: *Amentuloradula* Devos et al., *Cladoradula* Spruce, *Dactyloradula* Devos et al., *Metaradula* R.M.Schust., *Odontoradula* K.Yamada, *Radula*, and *Volutoradula* Devos et al. (Devos et al. 2011a; Patiño et al. 2017). However, the most recent revision by Renner et al. (2022), which incorporated two fossil *Radula* species preserved in Cretaceous Burmese amber to estimate divergence times, redefined the family by elevating *Dactyloradula* and *Cladoradula* to generic rank. Consequently, three genera are now recognized within Radulaceae and five subgenera are accepted in the genus *Radula* (Renner et al. 2022). Moreover, the genus *Radula* sensu lato has been the subject of extensive taxonomic and floristic research across various regions, resulting in numerous revisions and the description of many new species (e.g. Brown and Pócs 2001; Renner 2005, 2006, 2014, 2015; Renner et al. 2010, 2013a, 2013b, 2013c, 2014; So 2005a, 2005b, 2006; Singh et al. 2016a, 2016b; Oliveira-da-Silva and Ilkiu-Borges 2020; Oliveira-da-Silva et al. 2020, 2021; Gradstein 2021; Zhang and Zhu 2016; Zhang et al. 2021; Promma and Chantanaorrapint 2015; Promma et al. 2018; Pócs and Renner 2021).

During an ongoing of revision of Radulaceae in Southeast Asia, an interesting rheophyte *Radula* was collected from wet rocks and soil along the streamside in lowland evergreen forest from peninsular Thailand and Brunei Darussalam, parts of the Sundaland biodiversity hotspot. Morphological observations revealed a unique combination of characters not matching any previously described species. Here, we assess its taxonomic status using both morphological and molecular phylogenetic evidence, which together support its recognition as a new species.

## Materials and methods

### Morphological study

This study is based on recent collections from Thailand and Brunei. Voucher specimens of new species are deposited in HSNU and PSU herbaria. Morphological and anatomical characters were studied using stereo and light microscopes. The distinctive characters of the species were photographed using an Olympus BX43 microscope equipped with a DP71 digital camera and illustrated with Nikon ECLIPSE E200 with attached Nikon Y-IDT drawing tube after fully moistening samples in water. In addition, distribution and ecological data were compiled; brief descriptions and illustrations are provided.

### Taxon sampling

To analyze the phylogenetic placement of the new species, a total of 252 samples representing three genera of the family Radulaceae and five subgenera of the genus *Radula* currently recognized in Radulaceae were incorporated into a molecular data matrix. These samples were the ingroup in this study. *Frullania* sp., *Lepidolaena novae-zelandiae* (E.A.Hodgs. & S.W.Arnell) von Konrat et al., *Lepidolaena clavigera* (Hook.) Dumort. ex Trevis., *Porella navicularis* (Lehm. & Lindenb.) Pfeiff., and *Lejeunea tuberculosa* Steph. were employed as the outgroup, as these genera have been identified as close relatives of Radulaceae (Heinrichs et al. 2005; Forrest et al. 2006). Of these samples, 257 accessions were derived from previous studies focusing on the phylogeny of Radulaceae (Devos et al. 2011a, 2011b; Patiño et al. 2017; Renner et al. 2013b, 2022; Promma et al. 2025) and the GenBank database (https://www.ncbi.nlm.nih.gov/genbank/), including two samples of *R. rheophila* from Thailand. Herbarium voucher numbers and GenBank accession numbers used for phylogenetic analysis are provided in Suppl. material 2.

### DNA extraction, amplification and sequencing

The isolation of plant tissues and extraction of total DNA followed protocols previously used in the group (Promma et al. 2018, 2025; Shu et al. 2022). The total genomic DNA was extracted by using the DNAeasy plant mini kits (Qiagen, Hilden, Germany). Six chloroplast DNA markers, including *atp*B*-rbc*L, *psb*T*-psb*H, *psb*A*-trn*H, *rps*4, *trn*G, and *trn*L-F were sequenced by Jie Li Biology Inc., China (http://www.genebioseq.com)

The newly generated sequences were edited using PhyDE®0.997 (Müller et al. 2010), and a consensus sequence was assembled by comparison of the forward and reverse sequence of each individual. Alignment for the consensus sequences and published sequence data retrieved from GenBank was initially performed in MEGA v.11.0.13 (Tamura et al. 2021), and then checked by visual inspection and manually adjusted in PhyDE®0.997. Ambiguously aligned sites and gap-rich columns were excluded from phylogenetic analyses. Lacking parts of sequences and missing nucleotides were coded as missing data.

### Phylogenetic analyses

The phylogenetic trees of individual and combined datasets were constructed using Bayesian inference (BI) and maximum likelihood (ML) analysis. The individual marker sets and the combined dataset were first analyzed separately and compared for possible incongruence in the topology. As the trees showed no conflicting nodes, the datasets were combined. Bayesian inference analysis was performed using MrBayes v.3.2 (Ronquist et al. 2012). The best-fit model of evolution for each of the six plastid markers was identified by jModelTest 2 (Darriba et al. 2012) with the Akaike information criterion. This resulted in TVM+I+G model for *atp*B–*rbc*L, *psb*T–*psb*H, *rps*4, *trn*G and the concatenated six-marker dataset; GTR+I+G model for *psb*A–*trn*H; and TIM1+I+G model for *trn*L–F. The dataset was then analyzed using Markov chain Monte Carlo (MCMC) heuristic searches, performing four independent runs with four chains of 20 million generations, and trees were sampled every 1000 generations. A burn-in of 10% of the trees based on Tracer v.1.7.1 (Rambaut et al. 2018) was discarded before inferring a single majority-rule consensus phylogeny with Bayesian posterior probability (BI–PP) confidence values. Maximum likelihood (ML) analysis was performed using RAxML v.8 (Stamatakis 2014) with a single GTR+I+G model for the concatenated dataset, on the CIPRES Science Gateway (Miller et al. 2010). A rapid bootstrap analysis was conducted using the GTRGAMMAI substitution model with 1000 rapid bootstrapping replicates. Clades were considered supported if Bayesian posterior probability (BI–PP) ≥ 0.90 and maximum likelihood bootstrap percentages (ML–BS) ≥ 70%, sufficiently supported when BI–PP values ≥ 0.95 and ML–BS values ≥ 80%.

## Results

The concatenated dataset of *atp*B*–rbc*L, *psb*A*–trn*H, *psb*T*–psb*H, *rps*4, *trn*G, and *trn*L–F contains 3,466 characters (571, 554, 502, 590, 685, and 564 characters, respectively). Of the total characters, 2,021 are constant, 1,138 are parsimony informative, and 305 variable but parsimony uninformative.

**Figure 1.**
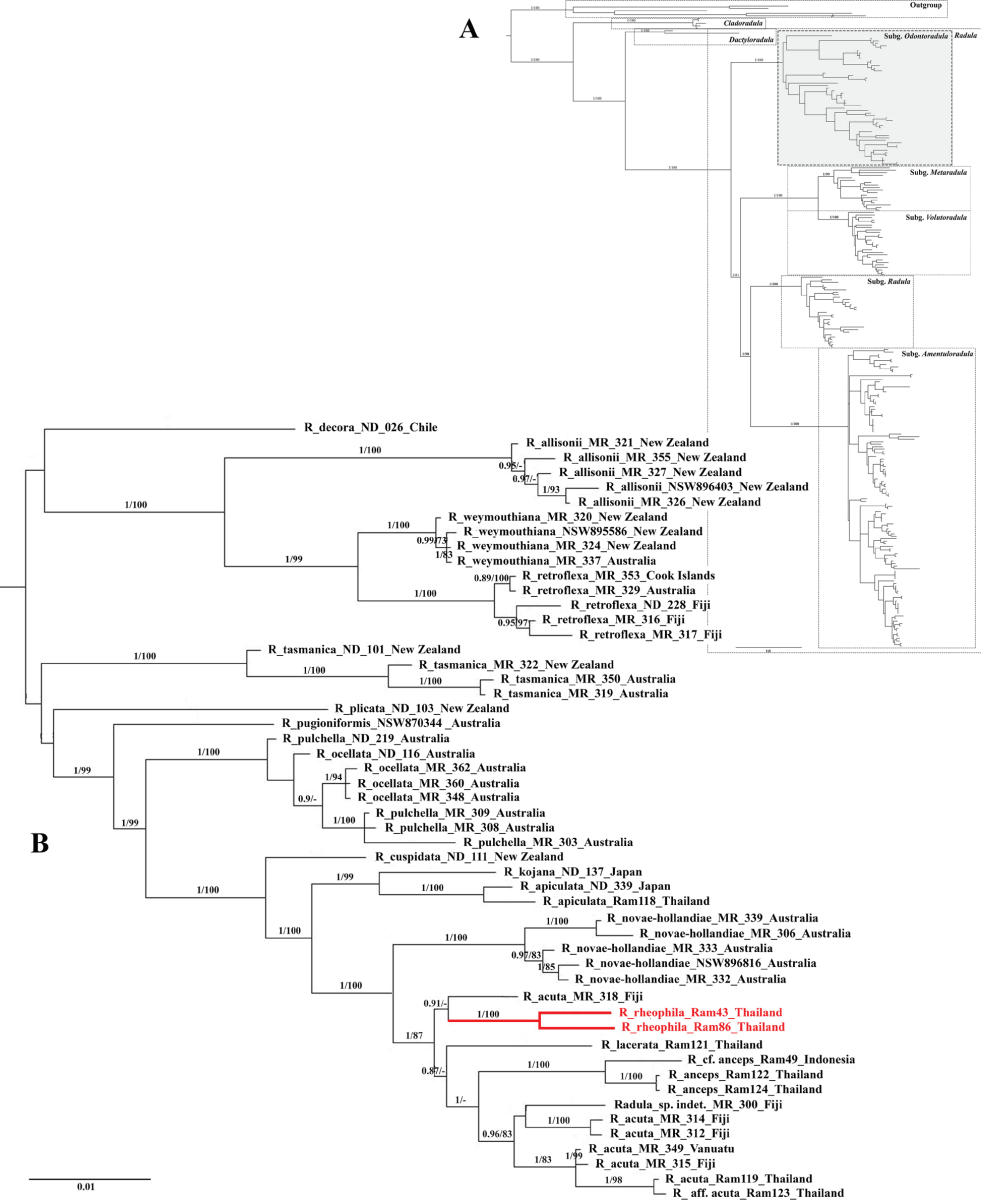
Majority-rule consensus tree based on Bayesian analyses of the combined dataset of *atpB–rbcL*, *psbA–trnH*, *psbT–psbH*, *rps4*, *trnG*, and *trnL–F*. Bayesian posterior probabilities values (BI–PP) and ML bootstrap values (ML–BS), are indicated at branches (BI–PP/ML–BS). **A**. Phylogenetic relationships of Radulaceae (see Suppl. material 1); **B**. Close up *Radula* subg. *Odontoradula*.

The tree topology of Radulaceae produced with BI and ML analyses resulted in congruent topologies with the relationships between three genera (*Radula*, *Cladoradula* (Spruce) M.A.M.Renner et al., and *Dactyloradula* (Devos et al.) M.A.M.Renner & Gradst.) and five subgenera of *Radula* corresponding to the previous publications (Devos et al. 2011a, 2011b; Patiño et al. 2017; Renner et al. 2022; Promma et al. 2025). The Bayesian consensus trees from the combined dataset are shown in Fig. 1 (1B enlarged from a group specified on 1A), with posterior probabilities (BI–PP ≥ 0.90) and maximum likelihood bootstrap (ML–BS ≥ 70) values plotted on the nodes.

The backbone phylogeny of the family Radulaceae is similar to that published and described by Devos et al. (2011a) and Renner et al. 2022. The newly tested taxon, *R. rheophila*, falls into a supported clade with Bayesian posterior probabilities values (BI–PP = 1, ML–BS = 100) (Fig. 1B) that is consistent with the *Radula* subg. *Odontoradula*. The two individuals of *Radula rheophila* formed a strongly supported clade (BI–PP = 1, ML–BS = 100), which is sister to *R. acuta* (MR_318) from Fiji; however, the relationship at the base of this subclade lacked strong support (0.91/–). Notably, *R. acuta* (MR_318) is paraphyletic to a monophyletic group comprising the remaining *R. acuta* entities.

### Taxonomic treatment

#### Radula rheophila

Taxon classificationPlantaeRadulalesRadulaceae

Promma & Chantanaorr. sp. nov.

F613211E-8205-5AD0-9E17-E1AA6B42E48D

Figs 2, 3

##### Type material.

Thailand. • Songkhla: Ton Nga Chang Wildlife Sanctuary, Ton Nga Chang Waterfall, lowland evergreen forest, 6°56.6937'N, 100°13.093'E, 483 m elev., on wet rocks along stream, 11 Feb 2018, *S. Chantanaorrapint 3045* (holotype: PSU!; isotypes: BKF!, HSNU!).

##### Diagnosis.

*Radula rheophila* resembles *R. novae-hollandiae* Hampe in lobule shape but differs in having ovate to broadly ovate leaf lobes with a broadly obtuse to rounded apex and entire margin.

##### Description.

***Plants*** yellowish-green to bright green when fresh, yellowish-brown in dry condition. Shoots up to 15 mm long, 1.56–2.30 mm wide; irregularly and densely pinnately branched, obliquely spreading; microphyllous and amentulose branches absent. ***Stems*** 156.5–280.0 µm diameter in transverse section, 8–14 cell rows across, with 40–80 rows of medullar cells, 24–30 rows of cortical cells, cortical cells as large as the medullary cells, 10–36 µm wide; cell walls pale brown to yellowish-brown, exterior cortical wall heavily and evenly thickened, interior cortical cell wall and medullary cell wall thin medially and thickened at the angles by concave trigones that make the cells circular in outline. ***Rhizoids*** brown, rhizoid-initial area convex. ***Leaf lobes*** loosely imbricate to slightly remote, widely spreading, ovate to broadly ovate, 0.75–1.10 mm long, 0.66–0.91 mm wide, apex broadly obtuse to rounded, margin entire, dorsal base strongly arched and slightly covering the stem to covering the stem ca. 1/2 of the stem-width in dorsal view. Leaf lobe marginal cells 7.5–17.5 × 12.5–17.5 µm, median cells 14.5–28.0 × 16.5–24.3 µm, basal cells 17.5–34.5 × 16.0–36.5 µm, thin-walled with indistinct or minute trigones; cuticle smooth. Oil bodies 2–3 per cell, grayish brown, ovoid or ellipsoidal, 10.9–16.4 × 7.0–9.3 µm, finely botryoidal. ***Leaf lobules*** remote, longitudinally rectangular or ligulate when large, broadly transversely rhombic or rectangular when small, 1/3–2/5 of the lobe-length, 375–678 mm long, 260–388 mm wide, apex obtuse or rarely emarginate, free exterior margin straight, or slightly sinuate, parallel or slightly inclined towards stem, free antical margin obliquely straight then arched to lobule free interior margin, free interior margin arched and slightly covering the stem to ca. 1/2 of the stem-width, line of insertions longitudinal with the stem, nearly straight or slightly arched, carinal region not or slightly inflated, keel straight to slightly sinuate, extending at angles of 40–50° with the stem, sinus wide or none. Slime papillae of leaf lobule usually 3, one situated on the lobule-apex, rounded to ovoid and sunken into a distinct notch, the other two near the base of the free interior margin of leaf lobule (on margin above the top of the stem insertion) at intervals, obovoid or short pyriform (sometimes nearly rounded). ***Asexual reproduction*** not seen. ***Sexuality*** dioicous (androecia not seen)?. ***Gynoecia*** terminal on main shoots and lateral branches, with 1–2 subfloral innovations; bracts in 2 or 2.5 pairs, often one below the subfloral innovations, subsymmetrical, or sometimes asymmetrical, imbricate, bract-lobe oblong-elliptic (upper) or ovate (lower), 1204–1264 µm long, 715–837 µm wide, apex rounded, slightly incurved, margin entire, bract-lobules ovate, one-thirds (lowest) to two-thirds (upper) the lobe area, apex obtuse to rounded, sometimes recurved, margin entire, keel slightly arched; perianths not seen. ***Sporophytes*** not seen.

**Figure 2.**
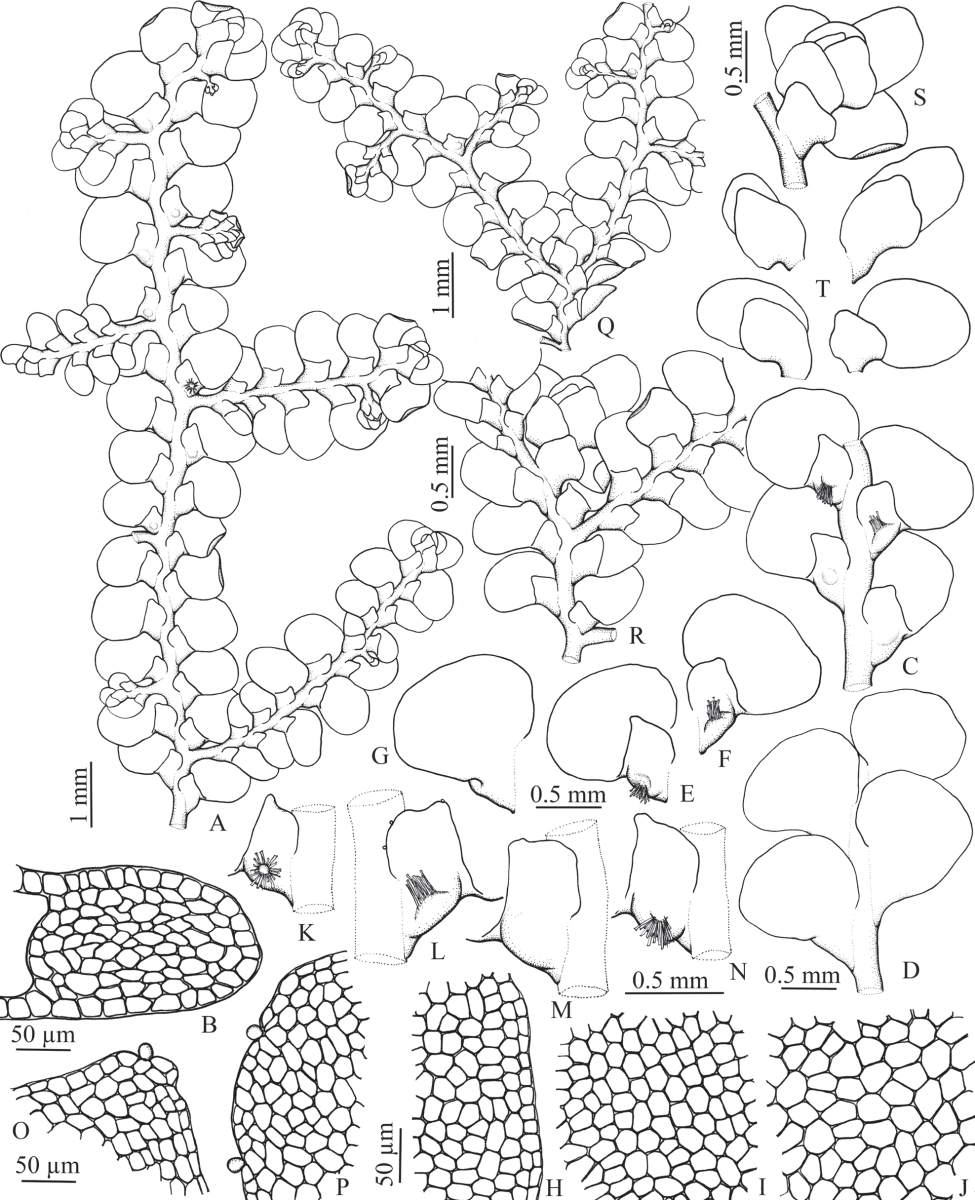
*Radula rheophila* Promma & Chantanaorr. **A**. Portion of sterile plant; **B**. Transverse section of stem; **C, D**. Portions of sterile plants; **C**. Ventral view; **D**. Dorsal view; **E–G**. Lateral leaves; **E, F**. Ventral views; **G**. Dorsal view; **H**. Marginal cells of leaf-lobe; **I**. Median cells of leaf-lobe; **J**. Basal cells of leaf-lobe; **K–N**. Leaf-lobules; **L**. with hyaline papillae; **O**. Apex of leaf-lobule with hyaline papilla; P. Free interior margin of leaf lobule with hyaline papillae; **Q, R**. Portions of female plants, with young gynoecia; **S**. Young gynoecium; **T**. Female bracts. Drawn by C. Promma; based on *S. Chantanaorrapint 3045* (holotype: PSU).

**Figure 3.**
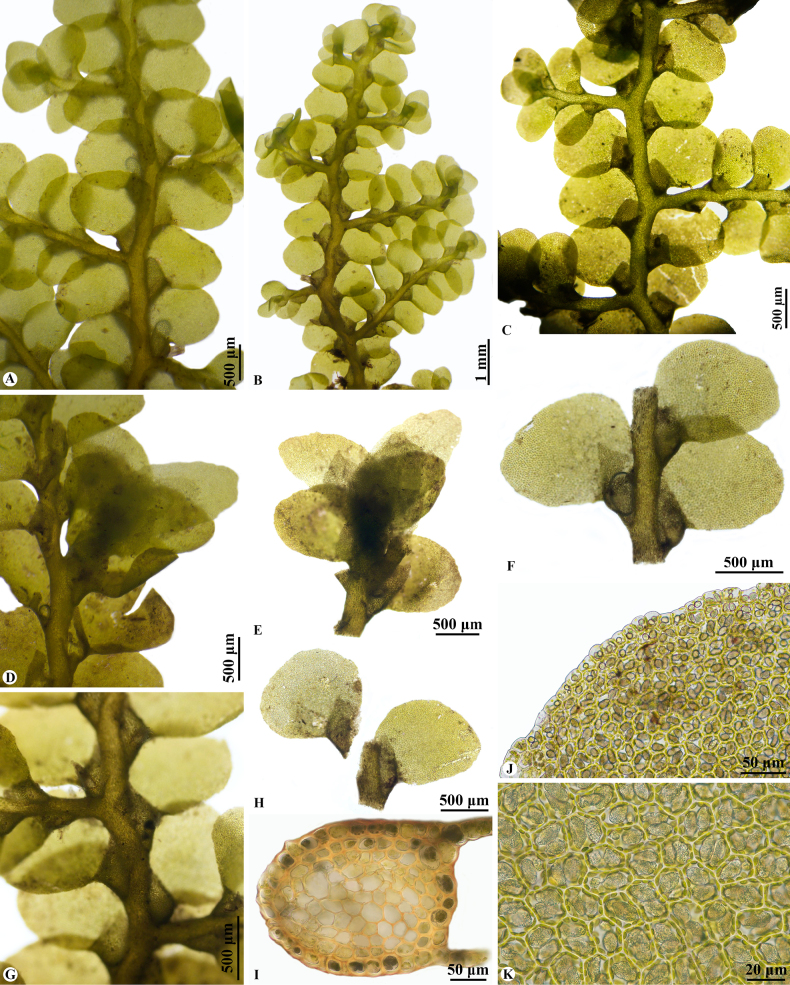
*Radula rheophila* Promma & Chantanaorr. **A–C**. Portions of sterile plants; **A**. Dorsal view; **B–C**. Ventral views; **D**. Portion of female plant, with young gynoecia; **E**. Young gynoecium; **F**. Portion of sterile plant, showing lateral leaves; **G**. Portion of sterile plant, showing leaf lobules; **H**. Lateral leaves; **I**. Transverse section of stem; **J**. Marginal cells of leaf lobe; **K**. Median cells of leaf lobe, showing oil bodies. Photographed by C. Promma; based on *S. Chantanaorrapint 3045* (holotype: PSU).

##### Etymology.

The specific epithet “*rheophila”* derives from the Greek *rheo-* meaning “flowing” and *-phila* meaning “loving,” referring to the species’ affinity for habitats associated with running water.

##### Habitat and ecology.

**Figure 4.**
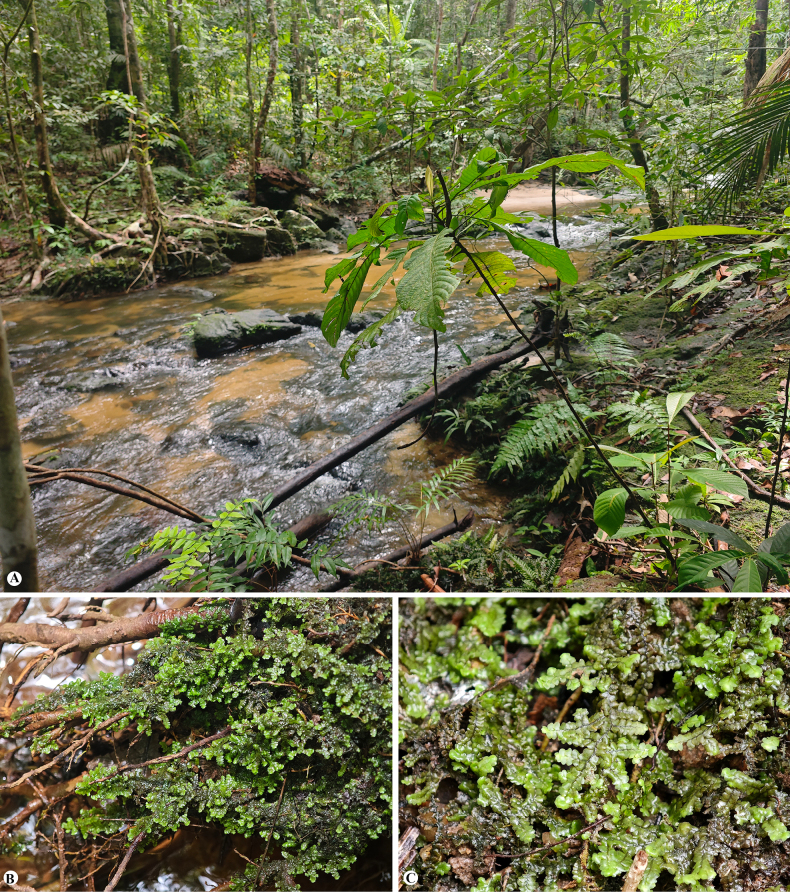
*Radula rheophila* Promma & Chantanaorr. **A**. Natural habitat at the type locality, growing on wet rocks, tree roots, and soil along streamside in lowland evergreen forest; **B**. Microhabitat showing plants creeping on roots over running water; **C**. Habit of the plant. Photographed by C. Promma.

*Radula rheophila* typically grows on wet rocks, roots, and soil along streamside in lowland evergreen forest, at elevations ranging from 75 to 490 m (Fig. 4A). The species prefers constantly moist and shaded microhabitats, often in association with other liverworts and mosses.

##### Distribution.

*Radula rheophila* is currently known from Thailand and Brunei. However, it may also occur in other areas in southern Thailand and Peninsular Malaysia as well, where similar ecological conditions prevail.

##### Additional specimens examined.

Thailand. • Songkhla: Ton Nga Chang Wildlife Sanctuary, Ton Nga Chang Waterfall, 6°56.7035'N, 100°13.147'E, 490 m elev., 21 Mar 2014, *S. Chantanaorrapint & C. Promma 3503* (HSNU, PSU: B01415), *S. Chantanaorrapint & C. Promma 3504* (HSNU, PSU: B01416); *S. Chantanaorrapint & C. Promma 3875* (PSU); • 6°56.6937'N, 100°13.093'E, 483 m elev., 11 Feb 2018, *S. Chantanaorrapint 3044* (HSNU, PSU); • 6°56.7243'N, 100°13.2778'E, 466 m elev., 21 Oct 2025, *C. Promma et al. 20251021-10*; • 6°56.7293'N, 100°13.2707'E, 463 m elev., 21 Oct 2025, *C. Promma et al. 20251021-11* (PSU); • 6°56.7295'N, 100°13.2758'E, 465 m elev., 21 Oct 2025, *C. Promma et al. 20251021-12* (PSU); • 6°56.6947'N, 100°13.0895'E, 478 m elev., 21 Oct 2025, *C. Promma et al. 20251021-24* (PSU). Brunei Darussalam. • Temburong: Kuala Belalong Field Studies Centre of University Brunei Darussalam, the opposite river, 4°32.8648'N, 115°9.4995'E, 78 m elev., 16 Dec 2015, *C. Promma 20151216-1* (HSNU).

## Discussion

*Radula rheophila* is distinguished by a unique combination of morphological characters, including the presence of 2 or 2.5 pairs of female bracts that typically differ in size; two pairs are positioned above the subfloral innovations, an additional single bract is often situated below the subfloral innovations. The leaf lobules are longitudinally rectangular or ligulate when large, and broadly transversely rhombic or rectangular when small, measuring one-third to two-fifths of the lobe length. The leaf lobes are ovate to broadly ovate, with a broadly obtuse to rounded apex and an entire margin. The oil bodies are finely botryoidal, occurring at two to three per cell. In transverse section, the stem exhibits heavily and evenly thickened exterior cortical cell walls, while the inner cortical and medullary cells are equal in size and shape, thin-walled, pale yellowish brown, and thickened at the angles by concave trigones that render the cells circular in outline.

Molecular phylogenetic analyses confirm that *Radula rheophila* belongs to *Radula* subg. *Odontoradula* and forms a sister relationship with *R. acuta* (MR_318) from Fiji. Together, this pair forms a clade sister to a broader group comprising the remaining *R. acuta* entities, *R. anceps* Sande Lac., and *R. lacerata* Steph., all of which are typical representatives of this subgenus in Thailand.

Subgenus *Odontoradula* was originally defined by Yamada (1979) for species possessing acute to apiculate or toothed leaves and two to four pairs of female bracts. Later molecular work revealed that this subgenus is morphologically heterogeneous, comprising species both conforming to and deviating from Yamada’s original circumscription (Devos et al. 2011a). The placement of *R. rheophila* within subg. *Odontoradula* is supported by its diagnostic morphology—particularly the presence of 2 or 2.5 pairs of female bracts, a distinct stem anatomy, and the presence of 2–3 oil bodies per cell. However, *R. rheophila* differs from other members of the subg. *Odontoradula* by its ovate to broadly ovate leaf lobes with broadly obtuse to rounded apices and entire margins.

Among the species of *Radula* subg. *Odontoradula*, *R. novae-hollandiae* Hampe (Australia and New Zealand; Renner et al. 2013b) is morphologically most similar to *R. rheophila* in its lobule morphology. Nevertheless, *R. novae-hollandiae* differs in having leaf lobes that are typically acute, only rarely obtuse at the apex. Based on molecular phylogenetic analyses, *R. rheophila* is closely related to *R. acuta* (MR_318) from Fiji (Fig. 1B). Furthermore, among the species found in Thailand, it is morphologically most similar to *R. acuta*. The two species share several features typical of the subg. *Odontoradula*, including entire-margined leaf lobes. However, *R. acuta* from Thailand differs in having apiculate or acute leaf lobes, subquadrate lobules approximately one-third the length of the lobe, and leaf cells with distinct triangular trigones.

## Supplementary Material

XML Treatment for Radula rheophila

## Additional information

### Use of AI

No use of AI was reported.

### Author ORCIDs

Chatchaba Promma https://orcid.org/0000-0001-5990-6251

Rui-Liang Zhu https://orcid.org/0000-0002-0163-8255

Sahut Chantanaorrapint https://orcid.org/0000-0002-9739-0994

### Data availability

All of the data that support the findings of this study are available in the main text or Supplementary Information.

## Supplementary materials

Supplementary material 1 Phylogenetic relationships of Radulaceae Data type tif https://binary.pensoft.net/file/1556296This dataset is made available under the Open Database License (http://opendatacommons.org/licenses/odbl/1.0/). The Open Database License (ODbL) is a license agreement intended to allow users to freely share, modify, and use this Dataset while maintaining this same freedom for others, provided that the original source and author(s) are credited. Chatchaba Promma, Rui-Liang Zhu, Sahut Chantanaorrapint

Supplementary material 2 Appendix S1 Data type docx Explanation note Voucher specimen information and GenBank accession numbers for specimens included in the molecular dataset: Ingroup/Outgroup: Species, Collector and Collection number, Herbarium, Source, DNA accession number, GenBank accession numbers for *atpB*–*rbcL*, *psbA*–*trnH*, *psbT*–*psbH*, *rps4*, *trnG*, and *trnL*–F. A dash (—) denotes missing data. https://binary.pensoft.net/file/1556297This dataset is made available under the Open Database License (http://opendatacommons.org/licenses/odbl/1.0/). The Open Database License (ODbL) is a license agreement intended to allow users to freely share, modify, and use this Dataset while maintaining this same freedom for others, provided that the original source and author(s) are credited. Chatchaba Promma, Rui-Liang Zhu, Sahut Chantanaorrapint
