# Supplementary material for: Radula rheophila, a new species of Radula (Radulaceae) from Thailand and Brunei Darussalam
Source: PhytoKeys. 2026 Mar 6;271:205–18. doi: 10.3897/phytokeys.271.181229 (PMC12988431; doi:10.3897/phytokeys.271.181229)
Supplement: Supplementary material 2 — Appendix S1 [file phytokeys-271-205_article-181229__-s002.docx]

**Appendix S1.** Voucher specimen information and GenBank accession numbers for specimens included in the molecular dataset: Ingroup/Outgroup: *Species*, Collector and Collection number, Herbarium, Source, DNA accession number, GenBank accession numbers for *atp*B*–rbc*L, *psb*A*–trn*H, *psb*T*–psb*H, *rps*4, *trn*G, and *trn*L–F. A dash (—) denotes missing data.

**Ingroup:** ***Cladoradula boryana*** (F.Weber) M.A.M.Renner et al., T. Pócs 88110/AR, E, Tanzania, ND_178, HM992024, HM992117, HM992200, HM992285, HM992375, —. ***Cladoradula boryana***, S. Ingram & K. Ferrell-Ingram 1765, Costa Rica, ND_060, HM991983, HM992076, HM992166, HM992245, HM992335, —. ***Cladoradula campanigera*** (Mont.) M.A.M.Renner et al., N. Ohnishi HIRO225, GOET, Japan, ND_042, HM991977, HM992070, HM992160, HM992239, HM992330, —. ***Cladoradula perrottetii*** (Gottsche ex Steph.) M.A.M.Renner et al., M. Mizutani NY15272, NY, Japan, ND_158, HM992018, HM992111, HM992194, HM992279, HM992369, —. ***Cladoradula tenax*** (Lindb.) M.A.M.Renner et al., P.G. Davison & M.L. Hicks 2946, DUKE, USA, ND_129, HM992009, HM992102, HM992186, HM992271, HM992360, —. ***Dactyloradula brunnea*** (Steph.) M.A.M.Renner & Gradst., Ohnishi s.n., KUN-B0030736, Japan, RAM16, OP477172, —, —, OP490854, —, OP491095. ***Dactyloradula brunnea***, N. Ohnishi H3196644, H, Japan, ND_001, HM991961, HM992054, HM992147, —, HM992315, HM992403. ***Radula acuminata*** Steph., T. Pócs 02102/AA, EGR, Vietnam, ND_227, HM992034, HM992127, HM992208, HM992295, HM992384, HM992463. ***Radula acuta*** Mitt., S. Chantanaorrapint & C. Promma 2155, PSU, Thailand, RAM119, OP477145, —, —, —, OP490944, OP491067. ***Radula acuta***, M.A.M. Renner 5503, NSW890194, Fiji, MR_318, KF495334, KX827524, —, —, KF495272, KF495393. ***Radula acuta***, M.A.M. Renner 5330, NSW889318, Fiji, MR_312, —, —, —, —, KF495267, KF495387. ***Radula acuta***, M.A.M. Renner 5349, NSW889366, Fiji, MR_314, —, —, —, —, KF495268, KF495389. ***Radula acuta***, M.A.M. Renner 5416, NSW889521, Fiji, MR_315, —, —, —, —, KF495269, KF495390. ***Radula acuta***, E.A. Brown s.n., NSW971056, Vanuatu, MR_349, —, —, —, —, KF495302, KF495418. ***Radula*** aff. ***acuta***, S. Chantanaorrapint & C. Promma 3822, PSU, Thailand, RAM123, OP477149, —, OP490748, OP490831, OP490948, OP491071. ***Radula acutiloba*** Steph., M.A.M. Renner 5889, NSW, Australia, NSW976716, KM220231, —, —, KX827585, KM220093, KM220162. ***Radula allisonii*** Castle, M.A.M. Renner 6264, NSW896403, New Zealand, MR_326, KF495342, —, —, KX827557, KF495280, KF495397. ***Radula allisonii***, M.A.M. Renner 6269, NSW896414, New Zealand, MR_327, —, —, —, —, KF495281, KF495398. ***Radula allisonii***, P.J. de Lange 10144 & M.A.M. Renner, NSW973432, New Zealand, MR_355, —, —, —, —, KF495307, KF495423. ***Radula allisonii***, M.A.M. Renner 6072, NSW895347, New Zealand, MR_321, —, —, —, —, KF495275, KF495395. ***Radula allisonii***, M.A.M. Renner 6264, NSW896403, New Zealand, MR_326, —, —, —, KX827557, KF495280, KF495397. ***Radula amentulosa*** Mitt., M.A.M. Renner 5378, NSW, Fiji, RAM23, OP477177, OP477280, —, OP490861, OP490978, OP491102. ***Radula amentulosa***, C.D. Kilgour 941, NSW, Malaysia, RAM50, OP477199, OP477299, OP490775, OP490882, OP491002, OP491126. ***Radula amentulosa***, TP1163/k, NSW, Fiji, RAM59, OP477206, —, —, OP490889, OP491009, OP491133. ***Radula amentulosa***, C.D. Kilgour 945, NSW, Malaysia, RAM72, OP477215, OP477312, OP490786, OP490898, OP491018, OP491142. ***Radula amentulosa***, C. Promma 20180210-6a, HSNU, Thailand, RAM85, OP477226, OP477320, OP490794, OP490909, OP491030, OP491154. ***Radula amentulosa***, Rui-Liang Zhu et al. 20160921-201, HSNU, Malaysia, RAM14, OP477170, OP477275, OP490762, OP490852, OP490970, OP491093. ***Radula amentulosa***, M.A.M. Renner 5369, NSW, Fiji, RAM25, OP477179, OP477282, —, OP490863, OP490980, OP491104. ***Radula anceps*** Sande Lac., S. Chantanaorrapint & C. Promma 1386, PSU, Thailand, RAM122, OP477148, —, —, OP490830, OP490947, OP491070. ***Radula anceps***, S. Chantanaorrapint & C. Promma 3550, PSU, Thailand, RAM124, OP477150, OP477256, OP490749, OP490832, OP490949, OP491072. ***Radula*** cf. ***anceps***, Rui-Liang Zhu et al. 20170518-54, HSNU, Indonesia, RAM49, OP477198, OP477298, —, OP490881, OP491001, OP491125. ***Radula aneurismalis*** (Hook.f. & Taylor) Gottsche et al., J.A. Curnow 4525, CBG, Australia, ND_126, HM992007, HM992100, —, HM992269, HM992358, —. ***Radula aneurismalis***, M.A.M. Renner 5999 & E.A. Brown, NSW880859, Australia, RAM89, OP477229, —, —, —, OP491033, OP491157. ***Radula ankefinensis*** Gottsche, S. & T. Pócs 04011/G, EGR, Kenya, ND_222, HM992032, HM992125, HM992207, HM992293, HM992382, HM992461. ***Radula antilleana*** Castle, S.R. Gradstein 9448, GOET, Costa Rica, ND_088, HM991992, HM992085, HM992175, HM992254, HM992343, HM992429. ***Radula apiculata*** Sande Lac. ex Steph., T. Yamaguchi 1731, BR, Japan, ND_339, HM992050, HM992143, —, HM992311, —, HM992478. ***Radula apiculata***, C. Promma 20180211-2, HSNU, Thailand, RAM118, OP477144, OP477254, OP490745, OP490827, OP490943, OP491066. ***Radula appressa*** Mitt., T. Pócs 90113/AH, EGR, Madagascar, ND_229, HM992036, HM992129, HM992209, HM992297, HM992386, HM992465. ***Radula aquilegia*** (Hook. f. & Taylor) Gottsche et al., A. chafer-Verwimp & Verwimp 26039, Herb. Schafer-Verwimp, Portugal, ND_078, HM991990, HM992083, HM992173, HM992252, HM992341, HM992427. ***Radula australiana*** K.Yamada, J.A. Curnow 5635, CBG, Australia, ND_119, HM992005, HM992098, HM992183, HM992267, HM992356, HM992442. ***Radula australis*** Austin, B. Shaw 6089, DUKE, USA, ND_299, HM992049, HM992142, HM992220, HM992310, HM992399, HM992477. ***Radula buccinifera*** (Hook. f. & Taylor) Gottsche et al., H. Streimann 54341, CBG, Australia, ND_127, HM992008, HM992101, HM992185, HM992270, HM992359, HM992444. ***Radula caledonica*** Steph., Paul Coulerie Cou104, NOU, New Caledonia, RAM53, OP477200, OP477300, OP490776, OP490883, OP491003, OP491127. ***Radula carringtonii*** J.B. Jack, A. Schafer-Verwimp & Verwimp 25734, Herb. Schafer-Verwimp, Portugal, ND_018, HM991969, HM992062, HM992153, HM992231, HM992323, HM992409. ***Radula cathayana*** Promma et al., Yu-Mei Wei et al. 20150703-22A, HSNU, China, RAM92, OP477232, OP477325, OP490799, OP490914, OP491036, OP491160. ***Radula cathayana***, Rui-Liang Zhu et al. 20150810-157, HSNU, China, RAM94, OP477233, OP477326, OP490800, OP490915, OP491037, OP491161. ***Radula cathayana***, Rui-Liang Zhu et al. 20150810-100A, HSNU, China, RAM95, OP477234, OP477327, OP490801, OP490916, OP491038, OP491162. ***Radula cathayana***, Rui-Liang Zhu et al. 20150810-140, HSNU, China, RAM100, OP477126, —, —, OP490809, OP490925, OP491048. ***Radula cathayana***, Rui-Liang Zhu et al. 20150810-154, HSNU, China, RAM101, OP477127, —, —, OP490810, OP490926, OP491049. ***Radula cavifolia*** Hampe ex Gottsche et al., C. Promma 20180210-18a, HSNU, Thailand, RAM80, OP477222, OP477316, OP490790, OP490905, OP491026, OP491150. ***Radula complanata*** (L.) Dumort., B. Shaw F915, DUKE, USA, ND_311, HM992043, HM992136, —, HM992304, HM992393, —. ***Radula constricta*** Steph., T. Koponen H3187494, H, China, ND_004, HM991963, HM992056, —, HM992225, HM992317, —. ***Radula cubensis*** K.Yamada, A. Schafer-Verwimp & M. Preussing 23532, Herb. Schafer-Verwimp, Ecuador, ND_068, HM991985, HM992078, HM992168, HM992247, HM992337, HM992422. ***Radula cuspidata*** Steph., M.A.M. Renner AK280588, AK, ND_111, New Zealand, HM992002, HM992095, —, HM992264, HM992353, HM992439. ***Radula decora*** Gottsche, I. Holz & Franzaring CH0060, GOET, Chile, ND_026, HM991973, HM992066, —, HM992235, HM992327, HM992413. ***Radula demissa*** M.A.M.Renner, A. Schafer-Verwimp & Verwimp 14336, Herb. Schafer-verwimp, New Zealand, ND_053, HM991979, HM992072, HM992162, HM992241, HM992332, HM992417. ***Radula ectophylla*** Promma et al., C. Promma & L.P. Ang 20180412-16a, HSNU, Malaysia, RAM141, OP477165, OP477270, —, OP490847, OP490965, OP491088. ***Radula ectophylla***, C. Promma & L.P. Ang 20180412-23, HSNU, Malaysia, RAM142, OP477166, OP477271, OP490759, OP490848, OP490966, OP491089. ***Radula ectophylla***, C. Promma & L.P. Ang 20180412-13, HSNU, Malaysia, RAM143, OP477167, OP477272, —, OP490849, OP490967, OP491090. ***Radula ectophylla***, Rui-Liang Zhu et al. 20160915-160A, HSNU, Malaysia, RAM13, OP477164, OP477269, OP490758, OP490846, OP490964, OP491087. ***Radula eggersii*** K.Yamada, A. Schafer-Verwimp & M. Preussing 23330/A, Herb. Schafer-Verwimp, Ecuador, ND_058, HM991982, HM992075, HM992165, HM992244, HM992334, HM992420. ***Radula episcia*** Spruce, S. Churchill, M. Serrano et al. MO23708, MO, Bolivia, ND_148, HM992015, HM992108, HM992191, HM992277, HM992366, HM992449. ***Radula fendleri*** Gottsche, A. Schafer-Verwimp & M. Preussing 23250/A, Herb. Schafer-Verwimp, Ecuador, ND_074, HM991987, HM992080, HM992170, HM992249, HM992339, HM992424. ***Radula flavifolia*** (Hook.f. & Taylor) Gottsche et al., M. von Konrat 6527, HSNU, Chile, RAM129, OP477155, OP477261, OP490754, OP490837, OP490954, OP491077. ***Radula floridana*** Castle, B. Shaw 6209, DUKE, USA, ND_323, HM992046, HM992139, HM992218, HM992307, HM992396, HM992474. ***Radula forficata*** M.A.M.Renner, M.A.M. Renner-2118, NSW, Australia, NSW878032, KF440561, —, KX827458, KX827552, KF440626, KF440490. ***Radula formosa*** (C.F.W.Meissn. ex Spreng.) Nees var. ***formosa***, S. Chantanaorrapint & O. Suwanmala 1508, PSU, Thailand, RAM42, OP477191, OP477292, OP490771, OP490875, OP490994, OP491118. ***Radula formosa*** var. ***formosa***, M.A.M. Renner 5506, NSW, Fiji, RAM60, OP477207, OP477305, OP490779, OP490890, OP491010, OP491134. ***Radula formosa*** var. ***formosa***, C. Promma 20180209-16b, HSNU, Thailand, RAM78, OP477220, OP477314, OP490788, OP490903, OP491024, OP491148. ***Radula formosa*** var. ***formosa***, C. Promma 20180210-16, HSNU, Thailand, RAM79, OP477221, OP477315, OP490789, OP490904, OP491025, OP491149. ***Radula formosa*** var. ***formosa***, C. Promma & L.P. Ang 20180412-17, HSNU, Malaysia, RAM110, OP477137, OP477248, OP490738, OP490820, OP490936, OP491059. ***Radula formosa*** var. ***formosa***, C. Kilgour_971, NSW, Malaysia, NSW979086, KX827519, KX827545, KX827487, KX827586, KX827496, KX827508. ***Radula formosa*** var. ***formosa***, Rui-Liang Zhu et al. 20160916-116, HSNU, Malaysia, RAM09, OP477125, OP477238, OP490728, OP490808, OP490924, OP491047. ***Radula formosa*** var. ***formosa***, Rui-Liang Zhu et al. 20170518-105, HSNU, Indonesia, RAM39, OP477188, OP477289, OP490768, OP490872, OP490991, OP491115. ***Radula formosa*** var. ***formosa***, C.D. Kilgour 971, NSW, Malaysia, RAM73, OP477216, —, —, OP490899, OP491019, OP491143. ***Radula formosa*** var. ***formosa***, Rui-Liang Zhu et al. 20150807-109, HSNU, China, RAM96, —, —, —, OP490917, OP491039, OP491163. ***Radula formosa*** var. ***formosa***, Rui-Liang Zhu et al. 20150807-121, HSNU, China, RAM97, OP477235, —, OP490802, OP490918, OP491040, OP491164. ***Radula formosa*** var. ***spicata*** (Mitt.) Promma et al., M.A.M. Renner 5612, NSW, Fiji, NSW980182, KX827520, KX827546, KX827488, KX827587, KX827497, KX827509. ***Radula formosa*** var. ***spicata***, M.A.M. Renner 5615-B, NSW1060324, Fiji, RAM68, OP477212, OP477309, OP490784, OP490895, OP491015, OP491139. ***Radula fulvifolia*** (Hook.f. & Taylor) Gottsche et al., T. Pócs s.n., EGR, Kenya, ND_215, HM992029, HM992122, HM992204, HM992290, HM992379, HM992458. ***Radula fulvifolia***, A. Schafer-Verwimp & M. Preussing 23443/A, Herb. Schafer-Verwimp, Ecuador, ND_019, HM991970, HM992063, HM992154, HM992232, HM992324, HM992410. ***Radula grandis*** Steph., D. Glenny CHR571846, CHR, New Zealand, ND_212, HM992028, HM992121, HM992203, HM992289, —, HM992457. ***Radula helix*** (Hook.f. & Taylor) Gottsche et al., B. Shaw 15676, HSNU, Chile, RAM125, OP477151, OP477257, OP490750, OP490833, OP490950, OP491073. ***Radula helix***, W. Buck 58590, HSNU, Chile, RAM126, OP477152, OP477258, OP490751, OP490834, OP490951, OP491074. ***Radula helix***, B. Shaw 14948, HSNU, Chile, RAM127, OP477153, OP477259, OP490752, OP490835, OP490952, OP491075. ***Radula helix***, B. Shaw 13201, HSNU, Chile, RAM128, OP477154, OP477260, OP490753, OP490836, OP490953, OP491076. ***Radula helix***, J.J. Engel 25665, HSNU, Chile, RAM116, OP477142, —, OP490743, OP490825, OP490941, OP491064. ***Radula helix***, M. von Konrat 6463, HSNU, Chile, RAM117, OP477143, OP477253, OP490744, OP490826, OP490942, OP491065. ***Radula hicksiae*** K.Yamada, M.A.M. Renner 6387, V.C. Linis & E.A. Brown, NSW, Australia, RAM31, OP477185, —, —, OP490869, OP490986, OP491110. ***Radula hicksiae***, J.A. Curnow & H. Streimann 3689, CBG, Australia, ND_120, HM992006, HM992099, HM992184, HM992268, HM992357, HM992443. ***Radula holtii*** Spruce, N. Devos & A. Vanderpoorten DV003, DUKE, Portugal, ND_281, HM992048, HM992141, HM992219, HM992309, HM992398, HM992476. ***Radula husnotii*** Castle, M.J. Lyon DB12895, MO, Costa Rica, ND_015, HM991968, HM992061, HM992152, HM992230, HM992322, HM992408. ***Radula imposita*** M.A.M.Renner, M.A.M. Renner 5275, NSW, Australia, NSW875821, KF432252, KX827523, KX827456, —, KF432393, KF440479. ***Radula inflexa*** Gottsche, A. Schafer-Verwimp & Verwimp 17830, Herb. Schafer-Verwimp, Dominica, ND_039, HM991976, HM992069, HM992159, HM992238, —, —. ***Radula japonica*** Gottsche, M. Higuchi 1198, BR, Japan, ND_353, HM992053, HM992146, HM992223, HM992314, HM992402, HM992481. ***Radula javanica*** Gottsche, P.J. de Lange, AK, New Zealand, AK323599, KM220207, —, KX827454, KX827547, KM220070, KM220137. ***Radula javanica***, T. Pócs s.n., EGR, Fiji, ND_220, HM992031, HM992124, HM992206, HM992292, HM992381, HM992460. ***Radula javanica***, S.R Gradstein & G. Dauphin DB12894, GOET, Costa Rica, ND_007, HM991964, HM992057, HM992148, HM992226, HM992318, HM992404. ***Radula jonesii*** Bouman et al., N. Devos s.n., DUKE, Spain, ND_267, HM992047, HM992140, —, HM992308, HM992397, HM992475. ***Radula jovetiana*** K.Yamada, M.A.M. Renner 6346, NSW, Australia, NSW896752, KF440585, KX827529, KX827463, —, KX827529, KF440517. ***Radula kojana*** Steph., M. Mizutani 14255, DUKE, Japan, ND_137, HM992013, HM992106, —, HM992275, HM992364, HM992447. ***Radula koponenii*** K.Yamada & Piippo, Rui-Liang Zhu et al. 20160916-44, HSNU, Malaysia, RAM15, OP477171, OP477276, —, OP490853, OP490971, OP491094. ***Radula lacerata*** Steph., S. Chantanaorrapint & C. Promma 2171, PSU, Thailand, RAM121, OP477147, —, OP490747, OP490829, OP490946, OP491069. ***Radula ligula*** Steph., E.L. Dos Santos 465, SP477636, Brazil, RAM30, OP477184, OP477286, OP490766, OP490868, OP490985, OP491109. ***Radula ligula***, Rui-Liang Zhu & Lei Shu 20171203-47, HSNU, Brazil, RAM114, OP477140, OP477251, OP490741, OP490823, OP490939, OP491062. ***Radula lindenbergiana*** Gottsche ex C.Hartm., A. Schafer-Verwimp & Verwimp 25732/A, Herb. Schafer-Verwimp, Portugal, ND_063, HM991984, HM992077, HM992167, HM992246, HM992336, HM992421. ***Radula lingulata*** Gottsche, S. Chantanaorrapint & C. Promma 1091, PSU, Thailand, RAM40, OP477189, OP477290, OP490769, OP490873, OP490992, OP491116. ***Radula lingulata***, S. Chantanaorrapint & C. Promma 1097, HSNU, Thailand, RAM112, OP477139, OP477250, OP490740, OP490822, OP490938, OP491061. ***Radula*** cf. ***lingulata*** Gottsche, Renner 5514 et al., NSW973388, Fiji, RAM56, OP477203, OP477302, OP490778, OP490886, OP491006, OP491130. ***Radula loriana*** Castle, M.A.M. Renner 6272, NSW, Australia, NSW970882, KF440604, —, KX827474, KX827573, KF440668, KF440538. ***Radula madagascariensis*** Gottsche, A. Szabó 9614/DV, EGR, Madagascar, ND_232, HM992037, HM992130, HM992210, HM992298, HM992387, HM992466. ***Radula marginata*** Gottsche et al., P.J. de Lange s.n. & M.A.M. Renner, NSW973389, New Zealand, RAM75, OP477217, —, OP490787, OP490900, OP491021, OP491145. ***Radula marginata***, M.A.M. Renner 6266, NSW896409, New Zealand, RAM22, OP477176, OP477279, OP490764, OP490860, OP490977, OP491101. ***Radula marojezica*** E.W.Jones, T. Pócs 90103/AE, EGR, Madagascar, ND_233, HM992038, HM992131, HM992211, HM992299, HM992388, HM992467. ***Radula mittenii*** Steph., M.A.M. Renner 6486, NSW, Australia, NSW897201, KF432305, KX827533, KX827467, KX827565, KF432391, KF440473. ***Radula*** cf. ***mittenii/reflexa*** Nees & Mont., Rui-Liang Zhu et al. 20170522-29a, HSNU, RAM46, Indonesia, OP477195, OP477295, OP490772, OP490878, OP490998, OP491122. ***Radula morobeana*** K.Yamada & Piippo, Rui-Liang Zhu et al. 20160921-73, HSNU, Malaysia, RAM104, OP477130, OP477241, OP490731, OP490813, OP490929, OP491052. ***Radula morobeana***, C. Promma & L.P. Ang 20180414-52, HSNU, Malaysia, RAM111, OP477138, OP477249, OP490739, OP490821, OP490937, OP491060. ***Radula morobeana***, Rui-Liang Zhu et al. 20160918-154, HSNU, Malaysia, RAM12, OP477156, OP477262, OP490755, OP490838, OP490955, OP491078. ***Radula morobeana***, C.D. Kilgour 952, NSW, Malaysia, RAM74, —, —, —, —, OP491020, OP491144. ***Radula multiamentula*** E.A.Hodgs., M.A.M. Renner AK280299, AK, New Zealand, ND_108, HM992001, HM992094, HM992182, HM992263, HM992352, HM992438. ***Radula multiamentula***, M.A.M. Renner 6195, NSW, New Zealand, RAM18, OP477174, —, —, OP490856, OP490973, OP491097. ***Radula multiamentula***, M.A.M. Renner 5897 & E.A. Brown, NSW, Australia, RAM24, OP477178, OP477281, —, OP490862, OP490979, OP491103. ***Radula myriopoda*** M.A.M.Renner, M.A.M. Renner 6580, NSW, Australia, NSW970376, KF440611, KX827536, KX827471, KX827570, KF440675, KF440545. ***Radula neotropica*** Castle, B. Allen NY11935, NY, Honduras, ND_160, HM992020, HM992113, HM992196, HM992281, HM992371, HM992452. ***Radula notabilis*** M.A.M.Renner, M.A.M. Renner 6505, NSW, Australia, NSW909500, —, KX827535, KX827469, KX827568, KF432378, KF440458. ***Radula novae-hollandiae*** Hampe, M.A.M. Renner 6362, NSW896816, Australia, MR_332, KF495348, —, —, KX827561, KF495286, KF495403. ***Radula*** ***novae-hollandiae***, NSW M.A.M. Renner 5261, NSW875807, Australia, MR_306, —, —, —, —, KF495261, KF495381. ***Radula*** ***novae-hollandiae***, M.A.M. Renner 5883, NSW898670, Australia, MR_339, —, —, —, —, KF495293, KF495410. ***Radula*** ***novae-hollandiae***, M.A.M. Renner 6362, et al., NSW896816, Australia, MR_332, —, —, —, KX827561, KF495286, KF495403. ***Radula*** ***novae-hollandiae***, M.A.M. Renner 6366, et al., NSW896820, Australia, MR_333, —, —, —, —, KF495287, KF495404. ***Radula nudicaulis*** Steph., A. Schafer-Verwimp & M. Preussing 23447, Herb. Schafer-Verwimp, Ecuador, ND_020, HM991971, HM992064, HM992155, HM992233, HM992325, HM992411. ***Radula nymanii*** Steph., M.A.M. Renner 6510, NSW, Australia, NSW898712, KF432310, KX827534, —, KX827566, KF432396, KF440478. ***Radula*** cf. ***obtusiloba*** Steph., Rui-Liang Zhu & C. Promma 20160728-21, HSNU, China, RAM17, OP477173, OP477277, OP490763, OP490855, OP490972, OP491096. ***Radula ocellata*** K.Yamada, J.A. Curnow 3664, CBG, Australia, ND_116, HM992003, HM992096, —, HM992265, HM992354, HM992440. ***Radula ocellata***, M.A.M. Renner 5090, et al., NSW970874, Australia, MR_348, —, —, —, —, KF495301, KF495417. ***Radula ocellata***, M.A.M. Renner 5091 & C. Kilgour, NSW973669, Australia, MR_360, —, —, —, —, KF495312, KF495428. ***Radula ocellata***, M.A.M. Renner 5053 & C. Kilgour, NSW973946, Australia, MR_362, —, —, —, —, KF495314, KF495430. ***Radula onraedtii*** K.Yamada, C. Promma & L.P. Ang 20170708-46, HSNU, China: Taiwan, RAM48, OP477197, OP477297, OP490774, OP490880, OP491000, OP491124. ***Radula oreopsis*** M.A.M.Renner, M.A.M. Renner 6581, NSW, Australia, NSW970377, KM220174, KM220104, KX827472, KX827571, KM220042, —. ***Radula ornata***, M.A.M. Renner 6469, V.C. Linis & E.A. Brown, NSW897016, Australia, RAM20, —, —, —, OP490858, OP490975, OP491099. ***Radula ornata*** E.A.Br. & Pócs, M.A.M. Renner 8389, NSW, Australia, RAM69, OP477213, OP477310, OP490785, OP490896, OP491016, OP491140. ***Radula pallens*** Nees & Mont., I. Holz CR000493, GOET, Costa Rica, ND_091, HM991994, HM992087, HM992177, HM992256, HM992345, HM992431. ***Radula pallens***, N. Salazar DB3609, GOET, Panama, ND_012, HM991966, HM992059, HM992150, HM992228, HM992320, HM992406. ***Radula patens***, M.A.M. Renner 6365, V.C. Linis & E.A. Brown, NSW, Australia, RAM21, OP477175, OP477278, —, OP490859, OP490976, OP491100. ***Radula patens*** K.Yamada, M.A.M. Renner 6348, V.C. Linis & E.A. Brown, NSW896765, Australia, RAM66, OP477210, —, OP490782, OP490893, OP491013, OP491137. ***Radula patens***, M.A.M. Renner 8102, NSW, Australia, RAM54, OP477201, OP477301, OP490777, OP490884, OP491004, OP491128. ***Radula physoloba*** Mont., M.A.M. Renner 6057B, NSW, New Zealand, RAM29, OP477183, OP477285, —, OP490867, OP490984, OP491108. ***Radula physoloba***, M.A.M. Renner 6056, NSW895322, New Zealand, RAM26, OP477180, —, —, OP490864, OP490981, OP491105. ***Radula physoloba***, M.A.M. Renner 6174, NSW895500, New Zealand, RAM132, OP477158, —, —, OP490840, OP490958, OP491081. ***Radula plicata*** Mitt., M.A.M. Renner AK280391, AK, New Zealand, ND_103, HM992000, HM992093, —, HM992262, HM992351, HM992437. ***Radula plumosa*** Mitt. ex Steph., J. Hyvönen DB3600, GOET, Argentina, ND_011, HM991965, HM992058, HM992149, HM992227, HM992319, HM992405. ***Radula pocsii*** K.Yamada, S. Churchill, M. Serrano et al. MO23444, MO, Bolivia, ND_150, HM992016, HM992109, HM992192, —, HM992367, HM992450. ***Radula polyclada*** A. Evans, B. Shaw F956, DUKE, USA, ND_315, HM992044, HM992137, HM992216, HM992305, HM992394, HM992472. ***Radula pseudoscripta*** M.A.M.Renner, M.A.M. Renner 01/73, AK, New Zealand, RAM134, OP477159, OP477264, —, OP490841, OP490959, OP491082. ***Radula pseudoscripta***, M.A.M. Renner 6170, NSW, New Zealand, NSW895495, KX827511, KX827525, KX827459, KX827554, KX827490, KX827499. ***Radula pseudoscripta***, M.A.M. Renner 6170, NSW895495, New Zealand, RAM27, OP477181, OP477283, OP490765, OP490865, OP490982, OP491106. ***Radula pseudoscripta***, M.A.M. Renner 6203, NSW, New Zealand, RAM55, OP477202, —, —, OP490885, OP491005, OP491129. ***Radula psychosis*** M.A.M. Renner, M.A.M. Renner 5090c, NSW, Australia, NSW970878, KF440592, —, KX827473, KX827572, KF440656, KF440526. ***Radula pugioniformis*** M.A.M.Renner, M.A.M. Renner 6841 & A.E. Orme, NSW, Australia, NSW870344, MF346528, MF346529, —, —, MF346530, MF346531. ***Radula pulchella*** Mitt., H. Streimann 63817, EGR, Australia, ND_219, HM992030, HM992123, HM992205, HM992291, HM992380, HM992459. ***Rudula pulchella***, Australia NSW M.A.M. Renner 5230 11 April 2011 NSW875776 MR_303, —, —, —, —, KF495259, KF495378. ***Rudula pulchella***, M.A.M. Renner 5276, NSW875822, Australia, MR_308, —, —, —, —, KF495263, KF495383. ***Rudula pulchella***, M.A.M. Renner 5304 & E.A. Brown, NSW877196, Australia, MR_309, —, —, —, —, KF495264, KF495384. ***Radula pusilla*** Spruce, A. Schafer-Verwimp & Verwimp 17767, Herb. Schafer-Verwimp, Dominica, ND_081, HM991991, HM992084, HM992174, HM992253, HM992342, HM992428. ***Radula quadrata*** Gottsche, T. Pócs, E.M. Kungu & A. Szabó 9230/S, EGR, Kenya, ND_225, HM992033, HM992126, —, HM992294, HM992383, HM992462. ***Radula queenslandica*** K.Yamada, J.A. Curnow 3846, CBG, Australia, ND_118, HM992004, HM992097, —, HM992266, HM992355, HM992441. ***Radula queenslandica***, M.A.M. Renner 6963 & T.C. Wilson, NSW870345, Australia, RAM28, OP477182, OP477284, —, OP490866, OP490983, OP491107. ***Radula ratkowskiana*** K.Yamada, M.A.M. Renner AK280205, AK, New Zealand, ND_102, HM991999, HM992092, HM992181, HM992261, HM992350, HM992436. ***Radula recubans*** Taylor, M. Burghardt DB21422, GOET, Mexico, ND_092, HM991995, HM992088, HM992178, HM992257, HM992346, HM992432. ***Radula recurvilobula*** K.Yamada, Paul Coulerie Cou85a, NOU, New Caledonia, RAM63, OP477209, OP477307, OP490781, OP490892, OP491012, OP491136. ***Radula resupinatiramosa*** Promma et al., L.P. Ang 20190331-12A, HSNU, Malaysia, RAM144, OP477168, OP477273, OP490760, OP490850, OP490968, OP491091. ***Radula rheophila*** Promma & Chantanaorr., S. Chantanaorrapint & C. Promma 3504, PSU, Thailand, RAM43, OP477192, —, —, —, OP490995, OP491119. ***Radula rheophila***, S. Chantanaorrapint 3045, PSU, Thailand, RAM86, OP477227, OP477321, OP490795, OP490910, OP491031, OP491155. ***Radula retroflexa*** Taylor, S. & T. Pócs 03281/C, EGR, Fiji, ND_228, HM992035, HM992128, —, HM992296, HM992385, HM992464. ***Radula retroflexa***, M.A.M. Renner 6311, et al., NSW896700, Australia, MR_329, —, —, —, —, KF495283, KF495400. ***Radula retroflexa***, P.J. de Lange CK183 & T.J. Martin, NSW973421, Cook Islands, MR_353, —, —, —, —, KF495305, KF495421. ***Radula retroflexa***, M.A.M. Renner 5428, NSW889533, Fiji, MR_316, —, —, —, —, KF495270, KF495391. ***Radula retroflexa***, M.A.M. Renner 5431, NSW889536, Fiji, MR_317, —, —, —, —, KF495271, KF495392. ***Radula robinsonii*** Steph., M.A.M. Renner 5250, NSW, Australia, NSW875795, KF440568, KX827522, KX827455, KX827548, KF440634, KF440501. ***Radula saccatiloba*** Steph., A. Schafer-Verwimp & Verwimp 18053, Herb. Schafer-Verwimp, Dominica, ND_075, HM991988, HM992081, HM992171, HM992250, HM992340, HM992425. ***Radula sainsburyana*** E.A.Hodgs. & Allison, M.A.M. Renner, AK282969, AK, New Zealand, ND_098, HM991996, HM992089, HM992179, HM992258, HM992347, HM992433. ***Radula sainsburyana***, M.A.M. Renner 5912 & E.A. Brown, NSW, RAM19, Australia, —, —, —, OP490857, OP490974, OP491098. ***Radula sainsburyana***, M.A.M. Renner 6085, NSW895360, New Zealand, RAM131, —, —, —, —, OP490957, OP491080. ***Radula scariosa*** Mitt., A. Schafer-Verwimp & Verwimp 18757/A, Herb. Schafer-Verwimp, Malaysia, ND_076, HM991989, HM992082, HM992172, HM992251, —, HM992426. ***Radula scariosa***, S. Chantanaorrapint & C. Promma 1323, PSU, Thailand, RAM02, —, —, —, —, —, OP491043. ***Radula scariosa***, S. Chantanaorrapint & C. Promma 2000, PSU, Malaysia, RAM35, —, —, —, —, OP490988, OP491112. ***Radula scariosa***, S. Chantanaorrapint & C. Promma 3741, PSU, Thailand, RAM36, —, —, —, —, OP490989, OP491113. ***Radula scariosa***, C.D. Kilgour 946, NSW, Malaysia, RAM62, OP477208, OP477306, OP490780, OP490891, OP491011, OP491135. ***Radula scariosa***, M.A.M. Renner 5640, NSW1060389, Fiji, RAM67, OP477211, OP477308, OP490783, OP490894, OP491014, OP491138. ***Radula scariosa***, C. Promma & L.P. Ang 20180412-25, HSNU, Malaysia, RAM107, OP477133, OP477244, OP490734, OP490816, OP490932, OP491055. ***Radula scariosa***, C. Promma & L.P. Ang 20180412-15, HSNU, Malaysia, RAM108, OP477134, OP477245, OP490735, OP490817, OP490933, OP491056. ***Radula scariosa***, M.A.M. Renner 5615-A, NSW, Fiji, RAM139, OP477163, OP477268, —, OP490845, OP490963, OP491086. ***Radula scariosa***, M.A.M. Renner 5593, NSW, Fiji, NSW895626, KX827512, KX827527, KX827460, KX827556, —, KX827500. ***Radula* sp.**, S. Churchill, M. Decker & F. Morgo MO22187, MO, Bolivia, ND_142, HM992014, HM992107, HM992190, HM992276, HM992365, HM992448. ***Radula* sp.**, T. Pócs, R.E. Magill & A. Rupf 9288/R, EGR, France, ND_234, HM992039, HM992132, HM992212, HM992300, HM992389, HM992468. ***Radula* sp.**, A. Schafer-Verwimp & Verwimp 23835, Herb. Schafer-Verwimp, Thailand, ND_045, HM991978, HM992071, HM992161, HM992240, HM992331, HM992416. ***Radula* sp.**, Rui-Liang Zhu et al. 20170522-8e, HSNU, Indonesia, RAM47, OP477196, OP477296, OP490773, OP490879, OP490999, OP491123. ***Radula* sp.** indet., NSW-Brown 05/362, NSW, Fiji, MR_300, —, —, —, —, KF495256, KF495375. ***Radula splendida*** M.A.M.Renner & Devos, P.J. de Lange 12134 & M.A.M. Renner, AK, New Zealand, RAM136, OP477161, OP477266, —, OP490843, OP490961, OP491084. ***Radula splendida***, M.A.M. Renner 6057A, NSW895332, New Zealand, RAM57, OP477204, OP477303, —, OP490887, OP491007, OP491131. ***Radula squarrosa*** K.Yamada, M.A.M. Renner 8450, NSW, Australia, RAM145, OP477169, OP477274, OP490761, OP490851, OP490969, OP491092. ***Radula squarrosa***, M.A.M. Renner 6381, NSW, Australia, NSW896832, KX827514, KX827531, KX827464, KX827562, —, KX827502. ***Radula squarrosa***, M.A.M. Renner 6453, V.C. Linis & E.A. Brown, NSW896990, Australia, RAM71, OP477214, OP477311, —, OP490897, OP491017, OP491141. ***Radula stenocalyx*** Mont., T. Pócs s.n., EGR, France, ND_235, HM992040, HM992133, HM992213, HM992301, HM992390, HM992469. ***Radula strangulata*** Hook.f. & Taylor, M.A.M. Renner AK280392, AK, New Zealand, ND_099, HM991997, HM992090, HM992180, HM992259, HM992348, HM992434. ***Radula subamentulosa*** Promma et al., M.A.M. Renner 5657, NSW1060433, Fiji, RAM58, OP477205, OP477304, —, OP490888, OP491008, OP491132. ***Radula subinflata*** Lindenb. & Gottsche, I. Holz & Schafer-Verwimp DB13093, GOET, Costa Rica, ND_030, HM991974, HM992067, HM992157, HM992236, HM992328, HM992414. ***Radula sullivantii*** Austin, B. Shaw 6189, DUKE, USA, ND_321, HM992045, HM992138, HM992217, HM992306, HM992395, HM992473. ***Radula tambuyukonensis*** Promma et al., C.D. Kilgour 966, NSW, Malaysia, RAM77, OP477219, OP477313, —, OP490902, OP491023, OP491147. ***Radula tasmanica*** Steph., M.A.M. Renner AK280184, AK, New Zealand, ND_101, HM991998, HM992091, —, HM992260, HM992349, HM992435. ***Radula tasmanica***, M.A.M. Renner 5935 & E.A. Brown, NSW895266, Australia, MR_319, —, —, —, —, KF495273, KF440509. ***Radula tasmanica***, M.A.M. Renner 5956, NSW972574, Australia, MR_350, —, —, —, —, KF495303, KF440510. ***Radula tasmanica***, M.A.M. Renner 6188, NSW895514, New Zealand, MR_322, —, —, —, —, KF495276, KF440548. ***Radula tenera*** Mitt., A. Schafer-Verwimp et al. 24230, Herb. Schafer-Verwimp, Ecuador, ND_022, HM991972, HM992065, HM992156, HM992234, HM992326, HM992412. ***Radula thiersiae*** K.Yamada, M.A.M. Renner 6461, NSW, Australia, NSW897007, —, —, KX827465, KX827563, KX827492, KX827503. ***Radula thiersiae***, M.A.M. Renner 6467, et al., NSW897014, Australia, RAM32, OP477186, OP477287, —, OP490870, OP490987, OP491111. ***Radula thiersiae***, M.A.M. Renner 8404, NSW, Australia, RAM90, OP477230, OP477323, OP490797, OP490912, OP491034, OP491158. ***Radula tjibodensis*** K.I.Goebel, A.L. Ilkiu-Borges et al. DB16663, GOET, Malaysia, ND_055, HM991980, HM992073, HM992163, HM992242, —, HM992418. ***Radula tokiensis*** Steph., T. Koponen H3187760, H, China, ND_003, HM991962, HM992055, —, HM992224, HM992316, —. ***Radula trapezoides*** Promma et al., S. Chantanaorrapint & C. Promma 1279A, PSU, Thailand, RAM05, —, —, —, OP490805, OP490921, OP491044. ***Radula trapezoides***, S. Chantanaorrapint & C. Promma 1211, PSU, Thailand, RAM06, —, —, —, OP490806, OP490922, OP491045. ***Radula trapezoides***, S. Chantanaorrapint & C. Promma 3622, PSU, Thailand, RAM07, OP477124, OP477237, OP490727, OP490807, OP490923, OP491046. ***Radula trapezoides***, C.D. Kilgour 957, NSW, Malaysia, RAM76, OP477218, —, —, OP490901, OP491022, OP491146. ***Radula trapezoides***, C. Promma 20180209-8a, HSNU, Thailand, RAM81, OP477223, OP477317, OP490791, OP490906, OP491027, OP491151. ***Radula trapezoides***, C. Promma 20180210-8a, HSNU, Thailand, RAM84, OP477225, OP477319, OP490793, OP490908, OP491029, OP491153. ***Radula trapezoides***, S. Chantanaorrapint & O. Suwanmala 1016, PSU, Thailand, RAM87, OP477228, OP477322, OP490796, OP490911, OP491032, OP491156. ***Radula trapezoides***, S. Chantanaorrapint & O. Suwanmala 1532a, PSU, Thailand, RAM91, OP477231, OP477324, OP490798, OP490913, OP491035, OP491159. ***Radula uvifera*** (Hook.f. & Taylor) Gottsche et al., M.A.M. Renner 02/167, AK, New Zealand, RAM135, OP477160, OP477265, —, OP490842, OP490960, OP491083. ***Radula vagans*** Steph., M. von Konrat 6471, HSNU, Chile, RAM115, OP477141, OP477252, OP490742, OP490824, OP490940, OP491063. ***Radula vagans***, W. Buck 59025, HSNU, Chile, RAM130, OP477157, OP477263, OP490756, OP490839, OP490956, OP491079. ***Radula verrucosa*** K.Yamada, C. Promma & L.P. Ang 20180412-16b, HSNU, Malaysia, RAM106, OP477132, OP477243, OP490733, OP490815, OP490931, OP491054. ***Radula verrucosa***, C. Promma & L.P. Ang 20180409-13b, HSNU, Malaysia, RAM137, OP477162, OP477267, OP490757, OP490844, OP490962, OP491085. ***Radula verrucosa***, Rui-Liang Zhu et al. 20160915-153, HSNU, Malaysia, RAM11, OP477146, OP477255, OP490746, OP490828, OP490945, OP491068. ***Radula voluta*** Taylor, A. Vanderpoorten AVW857, LG, UK: Wales, ND_014, HM991967, HM992060, HM992151, HM992229, HM992321, HM992407. ***Radula weymouthiana*** Steph., M.A.M. Renner 6201, NSW895586, New Zealand, MR_324, KF495340, —, —, KX827555, KF495278, KF495396. ***Radula weymouthiana***, M.A.M. Renner 6052 & E.A. Brown, NSW898459, Australia, MR_337, —, —, —, —, KF495291, KF495408. ***Radula weymouthiana***, M.A.M. Renner 6064, NSW895339, New Zealand, MR_320, —, —, —, —, KF495274, KF495394. ***Radula weymouthiana***, M.A.M. Renner 6201, NSW895586, New Zealand, MR_324, —, —, —, KX827555, KF495278, KF495396. ***Radula wichurae*** Steph., A. Schafer-Verwimp & Verwimp 26018, Herb. Schafer-Verwimp, Portugal, ND_057, HM991981, HM992074, HM992164, HM992243, HM992333, HM992419. ***Radula yangii*** K.Yamada, Rui-Liang Zhu et al. 20150811-64, HSNU, China, RAM98, OP477236, OP477323, OP490803, OP490919, OP491041, OP491165. ***Radula yangii***, C. Promma 20170403-45a, HSNU, China; Taiwan, RAM38, OP477187, OP477288, OP490767, OP490871, OP490990, OP491114. ***Radula yangii***, S. Chantanaorrapint & O. Suwanmala 966, PSU, Thailand, RAM45, OP477194, OP477294, —, OP490877, OP490997, OP491121. ***Radula yangii***, Rui-Liang Zhu et al. 20170518-115d, HSNU, Indonesia, RAM41, OP477190, OP477291, OP490770, OP490874, OP490993, OP491117. ***Radula yangioides*** Promma et al., S. Chantanaorrapint & C. Promma 3691, PSU, Thailand, RAM01, OP477123, —, —, OP490804, OP490920, OP491042. ***Radula yangioides***, Rui-Liang Zhu et al. 20160918-109, HSNU, Malaysia, RAM10, OP477136, OP477247, OP490737, OP490819, OP490935, OP491058. ***Radula yangioides***, S. Chantanaorrapint & O. Suwanmala 1621a, PSU, Thailand, RAM44, OP477193, OP477293, —, OP490876, OP490996, OP491120. ***Radula yangioides***, C. Promma 20180211-6, HSNU, Thailand, RAM83, OP477224, OP477318, OP490792, OP490907, OP491028, OP491152. ***Radula yangioides***, Rui-Liang Zhu et al. 20160922-206, HSNU, Malaysia, RAM102, OP477128, OP477239, OP490729, OP490811, OP490927, OP491050. ***Radula yangioides***, Rui-Liang Zhu et al.20160919-65A, HSNU, Malaysia, RAM103, OP477129, OP477240, OP490730, OP490812, OP490928, OP491051. ***Radula yangioides***, C. Promma 20180210-25, HSNU, Thailand, RAM105, OP477131, OP477242, OP490732, OP490814, OP490930, OP491053. ***Radula yangioides***, C. Promma & L.P. Ang 20180414-25, HSNU, Malaysia, RAM109, OP477135, OP477246, OP490736, OP490818, OP490934, OP491057.

**Outgroup:** ***Frullania* sp.**, B. Shaw F1086b/24, DUKE, South Africa, L1535, KF852106, KF851980, KF852263, —, —, —. ***Lejeunea tuberculosa*** Steph., D.G. Long 28596, E, Bhutan, E24, KF851996, JF513394, JF513410, JF513472, —, —. ***Lepidolaena clavigera*** (Hook.) Dumort. ex Trevis., John J. Engel 23052, F, New Zealand, IBC39, KF852058, AY607961, KF852194, AY608082, —, —. ***Lepidolaena novae-zelandiae*** (E.A.Hodgs. & S.W.Arnell) von Konrat et al*.*, von Konrat 99/Feb.#16, F, New Zealand, IBC32, —, AY607955, AY608012, AY608076, —, —. ***Porella navicularis*** (Lehm. & Lindenb.) Pfeiﬀ., Stotler & Crandall-Stotler 3410, ABSH, USA, IBC163, KF852055, AY507506, KF852186, AY507461, —, —.
